# Overexpression of soybean microRNA156b enhanced tolerance to phosphorus deficiency and seed yield in Arabidopsis

**DOI:** 10.1038/s41598-023-27847-2

**Published:** 2023-01-12

**Authors:** Guangyuan Lu, Zhitao Tian, Yifan Hao, Meihua Xu, Yongxin Lin, Jinxing Wei, Yongguo Zhao

**Affiliations:** 1 College of Biology and Food Engineering, Guangdong University of Petrochemical Technology, Maoming, 525000 People’s Republic of China; 2 College of Plant Science and Technology, Huazhong Agricultural University, Wuhan, 430062 People’s Republic of China

**Keywords:** Biotechnology, Genetics

## Abstract

microRNAs (miRNAs) are endogenous small RNAs that are key regulatory factors participating in various biological activities such as the signaling of phosphorus deficiency in the plant. Previous studies have shown that miR156 expression was modulated by phosphorus starvation in Arabidopsis and soybean. However, it is not clear whether the over-expression of soybean miR156b (*GmmiR156b*) can improve a plant’s tolerance to phosphorus deficiency and affect yield component traits. In this study, we generated Arabidopsis transgenic lines overexpressing *GmmiR156b* and investigated the plant’s response to phosphorus deficiency. Compared with the wild type, the transgenic Arabidopsis seedlings had longer primary roots and higher phosphorus contents in roots under phosphorus-deficit conditions, but lower fresh weight root/shoot ratios under either phosphorus-deficient or sufficient conditions. Moreover, the *GmmiR156b* overexpression transgenic lines had higher phosphorus content in shoots of adult plants and grew better than the wide type under phosphorus-deficient conditions, and exhibited increased seed yields as well as strong pleiotropic developmental morphology such as dwarfness, prolonged growth period, bushy shoot/branching, and shorter silique length, suggesting that the transgenic lines were more tolerant to phosphorus deficiency. In addition, the expression level of four *SQUAMOSA PROMOTER BINDING PROTEIN LIKE* (*SPL*) genes (i.e., *AtSPL4/5/6/15*) were markedly suppressed in transgenic plants, indicating that they were the main targets negatively regulated by *GmmiR156b* (especially *AtSPL15*) and that the enhanced tolerance to phosphorus deficiency and seed yield is conferred mainly by the miR156-mediated downregulation of *AtSPL15*.

## Introduction

Phosphorus (P) is an essential major nutrient required for plant growth and crop production^1^. It is a constituent element of many important molecules such as sugar phosphates, adenosine triphosphate, ribonucleic acids, deoxyribonucleic acid, and biomembrane phospholipids^2^. In addition, P also participates in many metabolic processes^3^. Although P is rich in the soils, only a small proportion (< 30%), in form of orthophosphate, can be uptake by plants^4,5^. Therefore, P deficiency is not uncommon and is a constraint for crop production in > 30% of the arable land that can be treated with P fertilizers^6,7^. The demand for P fertilizers worldwide in 2022 was predicted as high as 49.1 million tons^8^; however excessive application of P fertilizer also caused severe problems such as environmental pollution^7^. Therefore, the development of a new crop variety with efficient P use is required for agricultural production in P-deficit soils^9,10^.

Several sophisticated strategies have been evolved in plants to cope with low P stress^11^, such as growing more roots to promote P absorption, secreting acid phosphatase to hydrolyze P in the soil, and activating a cassette of phosphate stress-responsive genes to enhance the transport and absorption of P^12^. Several metabolic pathway genes, regulatory factors, membrane transporter proteins, and endogenous phytohormones are involved in P deficiency responses or P signal transduction^13^. Among these important factors, microRNAs (miRNAs) were regarded as key regulators in plant P starvation response^14–16^.

miRNAs are defined as a group of regulatory noncoding RNAs with 20–24 nucleotides in length, which modulate gene expression at the posttranscriptional level in plants. Among these, miR156 is the first kind of miRNA identified, which mainly targets genes that encode SQUAMOSA PROMOTER BINDING PROTEIN-LIKE (SPL) proteins^17^. By interacting with a subset of SPLs, miR156 exerts its function in a variety of developmental processes such as stress response^17,18^. For instance, miR156 together with its target SPL3 is a key regulator in response to P starvation by directly modulating the expression of three genes (i.e., *PLDZ2*, *Pht1;5*, and *miR399f*) related to P uptake in the model plant Arabidopsis^19^. The role of miRNAs in P-deficiency signal transduction was also investigated in rapeseed^20^ and common beans^21^. Apart from miR156, many other miRNAs were also detected in response to P starvation. For instance, miR156/778/827/2111 were induced upon P deprivation, while miR169/395/398 were suppressed^20–23^. In soybean, at least 57 miRNAs were significantly up- or down-regulated under P deficiency^24^.

Soybean is the major oil crop worldwide providing oil for humans and protein for livestock, with a total production of 362 million tons in 2020 (https://www.fao.org/faostat/en/#search/soybean). Under low P conditions, the production and quality of soybean are seriously affected. Recently, *miR156* was found to be up-regulated in Arabidopsis root and soybean leaf in response to low P stress^23,24^. Moreover, we also observed increased expression of *miR156b* in the shoots of soybean at 0.5–12 h after P-deficiency treatment (unpublished). However, very little is known about the consequence of overexpressing miR156 on plant architecture and seed yield under P stress conditions. To gain a deeper understanding of the function of miR156 in the phosphorus signaling pathway, we developed Arabidopsis lines overexpressing the soybean homolog of miR156b (termed *GmmiR156b* hereafter) and investigated their responses to P deficiency. The transgenic Arabidopsis lines overexpressing *GmmiR156b* were found to be more tolerant to P deficiency and have a higher seed yield. In addition, pleiotropy was also observed in the transgenic lines such as dwarfness, longer growth period, bushy shoot, and shorter silique length. Our study will shed new light on molecular mechanisms of P starvation adaption in soybean and will help find new ways to increase P use efficiency.

## Plant materials and methods

### *GmmiR156b* gene isolation, vector construction, and plant transformation

A genomic fragment comprising the whole sequence of *GmmiR156b* was amplified from soybean variety ‘BX10’ using the primer pair 156b-F_1_ and 156b-R_1_, which were designed based on the upstream and downstream of *GmmiR156b* (MI0001790, chr14: 990334-990453, http://www.mirbase.org), respectively (Supplementary table 1), and deposited in NCBI under accession No. OP784762. PCR was performed using the following parameters: one cycle of denaturing at 94 ℃ for 2 min; then 35 cycles of denaturing at 94 ℃ for 30 s, annealing at 58 ℃ for 30 s, and extension at 72 ℃ for 30 s; followed by inoculation at 72 ℃ for 5 min. The resultant PCR product was purified and inserted into the pCAMBIA1300-based T-DNA vector pJG045 driven by two 35S cauliflower mosaic virus promoters^25^. The vector was then induced into *Agrobacterium* strain GV3101 before transforming into *Arabidopsis thaliana* ecotype Columbia (Col-0) by the floral dipping method^26^. Seeds harvested from the transgenic generation 1 (T_1_) plants were screened for hygromycin resistance (25 µg mL^−1^) on Murashige and Skoog (MS) medium, and the survived plants were grown in separate pots to harvest T_2_ seeds, from which T_2_ lines exhibiting a 3:1 segregation ratio of hygromycin resistance were selected and further confirmed by PCR to be homozygous for insertion. The confirmed homozygous T_3_ lines were used for further analyses. The transcript abundance in transgenic lines was quantified by RT-PCR using the specific primer pairs 156b-F_1_ and 156-R_1_.

### Plant materials and growth conditions

The seeds of Col-0 and the *GmmiR156b* overexpression (OE) lines were sterilized with 10% sodium hypochlorite (v/v) for 10 min and washed 5 times with ddH_2_O, then germinated in Petri dishes containing MS solid medium (pH 5.8), 0.8% (w/v) agar, and 1% (w/v) sucrose. The seeds in Petri dishes were placed at 4 ℃ for 2 days for stratification and then moved to a plant growth chamber for germination 22 ℃ under long-day conditions (16 h light/8 h dark; 95 μmol m^−2^ s^−1^). After 5 days of growth, the seedlings were transferred to P-sufficient (+ P; 1 mmol L^−1^ KH_2_PO_4_) or P-deficient (−P; 0.5 mmol L^−1^ K_2_SO_4_) medium^27^. For seedlings analysis, seeds were germinated and then vertically grown on the surface of the agar. For adult plant analysis, the 5-day-old seedlings were transplanted from MS medium to vermiculite-filled pots supplied with + P or − P liquid medium, and grown in a greenhouse at a constant temperature of 22 ℃, photoperiod of 16 h light/8 h dark, and relative humidity of 70%.

### RT-PCR and qPCR analysis

Total RNA was prepared from fresh tissue samples using a Trizol reagent kit (Invitrogen, Carlsbad, CA, USA) and treated with DNaseI (Ambion, USA) to remove any genomic DNA residual. For RT-PCR analysis, 2 µg of total RNA was reversely transcribed by MMLV (Promega, USA) and oligo-d(T) primer to synthesize the first-strand cDNA following the manufacturer’s instruction. Then the ten-fold diluted cDNA was used as the template for the performance of RT-PCR with gene-specific primer pairs 156b-F_1_ and 156b-R_1_. Quantitative real-time PCR was carried out using primer combination 156b-F_2_/156b-R_2_ as previously reported^27^. The sequences of all primers for RT-PCR and quantitative real-time PCR in this study were given in Additional File 1. The relative gene expression levels were normalized by using *Tublin* as an internal reference gene following the method reported by Livak and Schmittgen^28,29^.

### Quantification of phosphorus content

The P content was measured by the malachite green method^30^. The shoots and roots of 10-day-old Arabidopsis seedlings, as well as shoots of adult plants at the bolting stage for Col-0, OE1, and OE2 lines growing in + P or − P treatments were excised, weighed, and dehydrated at 80 ℃ for 24 h. After digestion with sulfuric acid and hydrogen peroxide, approximately 40 µL of 5 mol L^−1^ H_2_SO_4_ per 20 mg dried samples were mixed with 3 mL of water, then first filtered by Whatman No. 4 filter paper, and then the supernatant aliquot was brought to a final volume of 1.5 mL with water, followed by the addition of 0.5 mL malachite green solution and mixed thoroughly. After inoculation at room temperature for at least 30 min, the phosphorus content was quantified from the optical absorbance values of the above mixture detected at a wavelength of 650 nm in a spectrophotometer (METASH, Shanghai, China).

### Measurement of biological traits

Fifteen seedlings per line were selected as one replicate for each treatment and four biological replicates were carried out. The root length of 10-day-old seedlings of Col-0, OE1, and OE2 lines growing in + P and − P conditions were investigated. Then the roots and shoots were dissected, weighted, and measured for P content. The fresh weight shoot/root ratio was calculated (Sha et al.^27^). For the investigation of yield component traits, each of the ten plants of Col-0 and OE1, and OE2 growing in either + P or − P conditions were collected with four biological replicates. Silique length was measured on five siliques from the medial inflorescence of a plant. Comparisons of means for all traits with replicates were statistically analyzed in IBM SPSS Version 16 (SPSS, Chicago, IL, USA).

### Ethics declaration

All authors agree to the ethics and consent to participate in this article and declare that this submission follows the policies of Scientific Reports. The paper is not being considered for publication elsewhere. All authors confirmed that experimental research on plants, including gene transformation and sample collection activities, complying with relevant institutional, national, and international guidelines and legislations. Furthermore, the handling of genetically modified organisms was carried out according to the local relevant guidelines and regulations of China.

## Results

### Construction and overexpression of *GmmiR156b*

**Figure 1:**
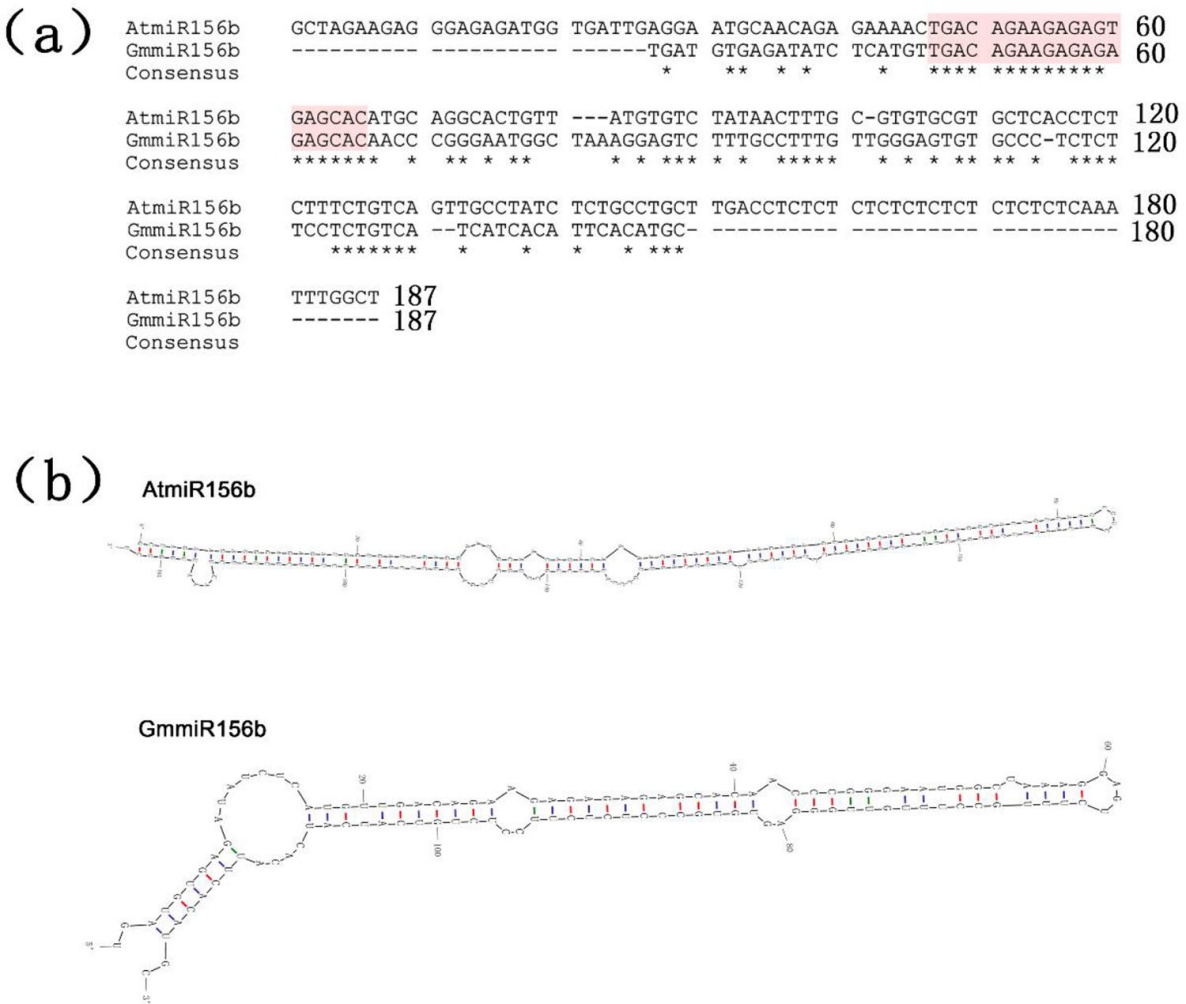
Comparison of *miR156b* in Arabidopsis and soybean. (**a**) DNA sequence alignment of miR156b precursors from Arabidopsis (*AtmiR156b*) and soybean (*GmmiR156b*). The mature gene sequences were highlighted in pink. (**b**) Secondary structure of *miR156b*s.

**Figure 2:**
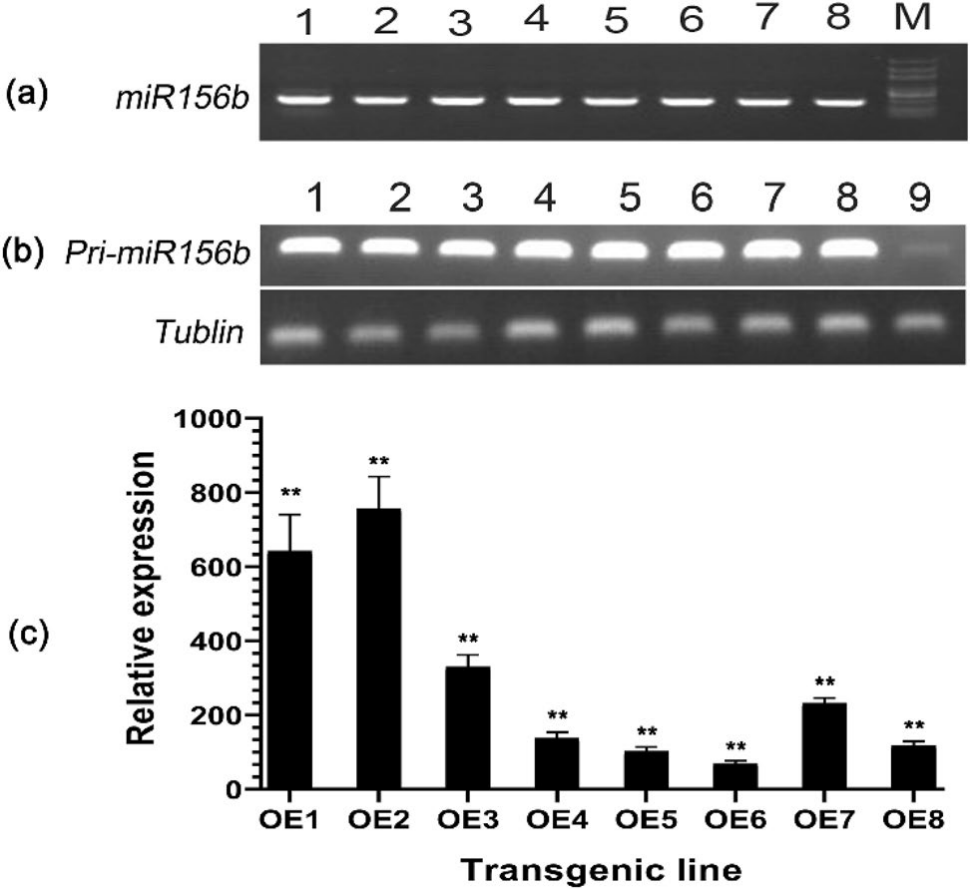
Verification of transgenic events and quantification of *GmmiR156b* expression in Arabidopsis seedlings. (**a**) PCR confirmation of positive transgenic Arabidopsis plants carrying *GmmiR156b* by gene-specific primers. (**b**) RT-PCR analysis of *pri-GmmiR156b* in the leaf of the wild type (WT) and eight independent transgenic lines. (**c**) Quantitative RT-PCR analysis of mature *GmmiR156b* in 8 overexpression (OE) lines. The relative expression level of mature *GmmiR156b* was normalized to that of the inner reference gene *Tublin*. Lanes 1–8, transgenic lines; lane 9, the WT. M, DNA marker DL2000. ** Indicates statistical difference between *GmmiR156b* and reference gene at *P* < 0.01. Original gels are presented in Supplementary Fig. 1.

A genomic fragment of pri-miR156b (*GmmiR156b*) was successfully amplified from soybean by PCR, which forms a typical stem-root structure showing 45% sequence similarity with Arabidopsis pri-miR156b but nearly identical in the mature gene sequences (Fig. 1). The DNA fragment was then inserted into the expression vector pJG045. After verification by DNA sequencing, the construct was transformed into Arabidopsis, and eight independent transgenic events were obtained. The T_1_ plants showing hygromycin resistance could be verified by PCR using gene-specific primers (Fig. 2a), then the expression level of *pri-GmmiR156b* was detected by RT-PCR, which indicated that all of the eight transgenic overexpression lines (named OE1 to OE8) exhibited a very high expression level of *GmmiR156b* (Fig. 2b). Next, quantitative real-time PCR (qPCR) further revealed that, if compared to the wild type (WT), the mature *GmmiR156b* was highly expressed in the eight transgenic lines, with a relative gene expression level varying from 70.0 to 756.3 folds increase and a mean of 298.9 folds for all OE lines (Fig. 2c). Of these, only two lines (namely OE1 and OE2) showed an overexpression higher than 600 folds, which were thus chosen for further analysis.

### Overexpression of *GmmiR156b* confers enhanced tolerance to P deficiency

**Figure 3:**
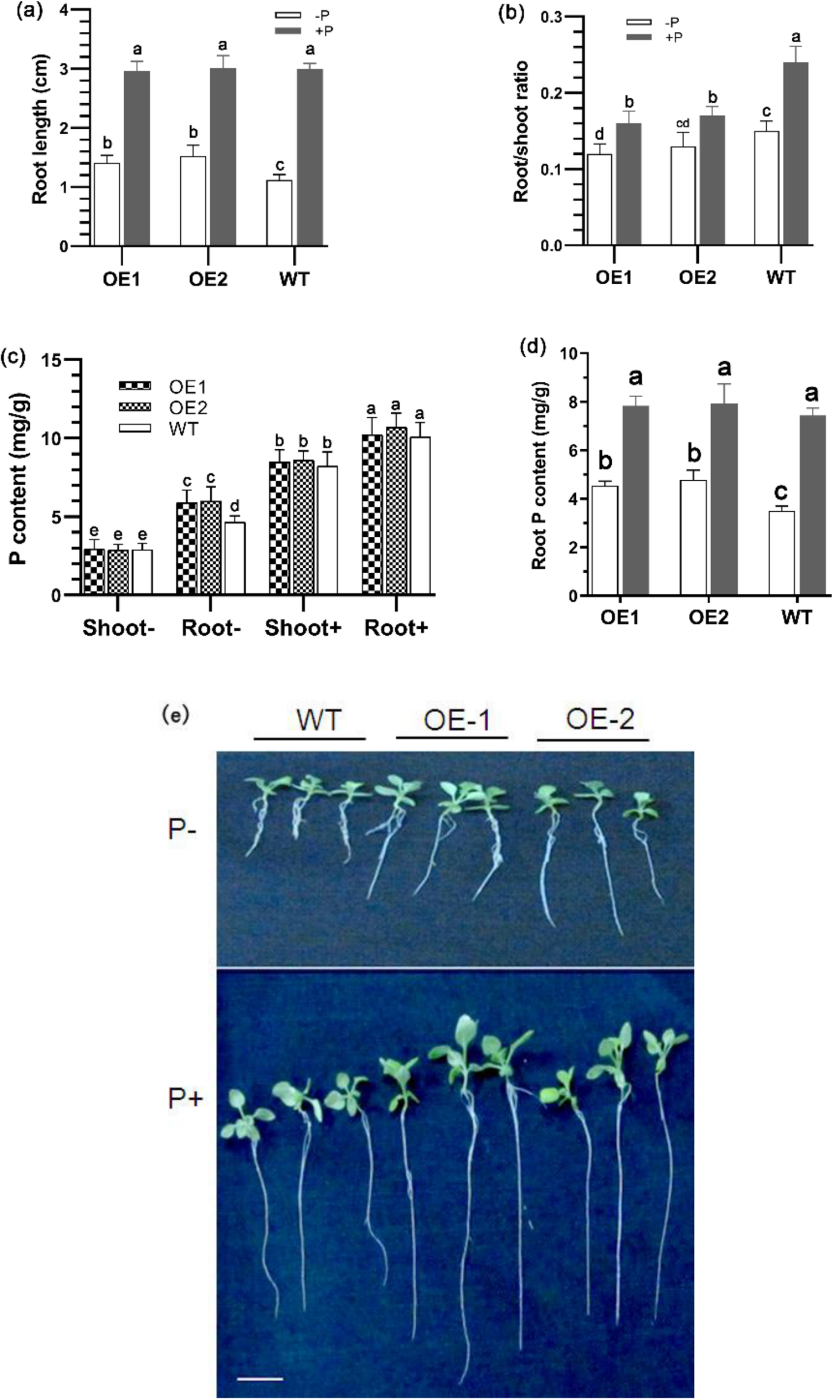
Root length, root/shoot ratio, and phosphorus contents for 10-day-old Arabidopsis seedlings and adult plants. (**a**) Root length of wild-type (WT) and transgenic plants overexpressing *GmmiR156b* (OE1, OE2) under + P (Pi-sufficient) and − P (Pi-deficient) conditions. (**b**) Fresh weight root/shoot ratio of 10-day-old seedlings of OE1, OE2, and WT under + P and − P conditions. (**c**) Phosphorus content in root and shoot of OE1, OE2, and WT under + P and − P conditions. (**d**) Phosphorate content in roots of adult plants of OE and WT lines. (**e**) The phenotype of WT and OE seedlings grown under + P and − P conditions. Different letters above the bars indicate statistical significance (*P* < 0.05). Shoot-, shoots from low P grown plant; Root-, roots from low Pi grown plant; Shoot +, shoots from high P grown plant; Root +, roots from high P grown plant. Scale bar = 1 cm.

At the seedling stage under + P condition, the Arabidopsis WT, OE1, and OE2 plants grew normally, all having primary roots with a similar length of 3 cm. However, under − P condition, the primary root length was reduced by nearly 50% for all lines, suggesting that P starvation limited plant growth substantially. Nevertheless, the roots of OE1 and OE2 (~ 1.5 cm) were significantly longer than that of WT (1.1 cm) under P nutritional stress (Fig. 3a), implying that plant growth could be partially rescued by overexpressing *GmmiR156b.* Meanwhile, the root/shoot ratios ranged from 0.15 to 0.24 under the + P condition but decreased markedly (0.12–0.15) under the − P condition, with the lowest value observed in OE1 and the highest in WT, under both situations (Fig. 3b). Interestingly, the higher root/shoot ratios in WT seedlings were found to be caused by higher fresh weight of roots. In addition, the total P contents of OE1 and OE2 lines were higher than that of WT in the roots but similar in the shoots under − P condition (Fig. 3c), showing that overexpressing *GmmiR156b* could promote the uptake and accumulation of P in the root rather than the shoot. When grown in normal condition, the total P contents of a given tissue (root or shoot) was similar among the OE lines and WT but varied between tissues, i.e., higher content in the roots and lower in the shoots at the seedling stage (Fig. 3c).

**Figure 4:**
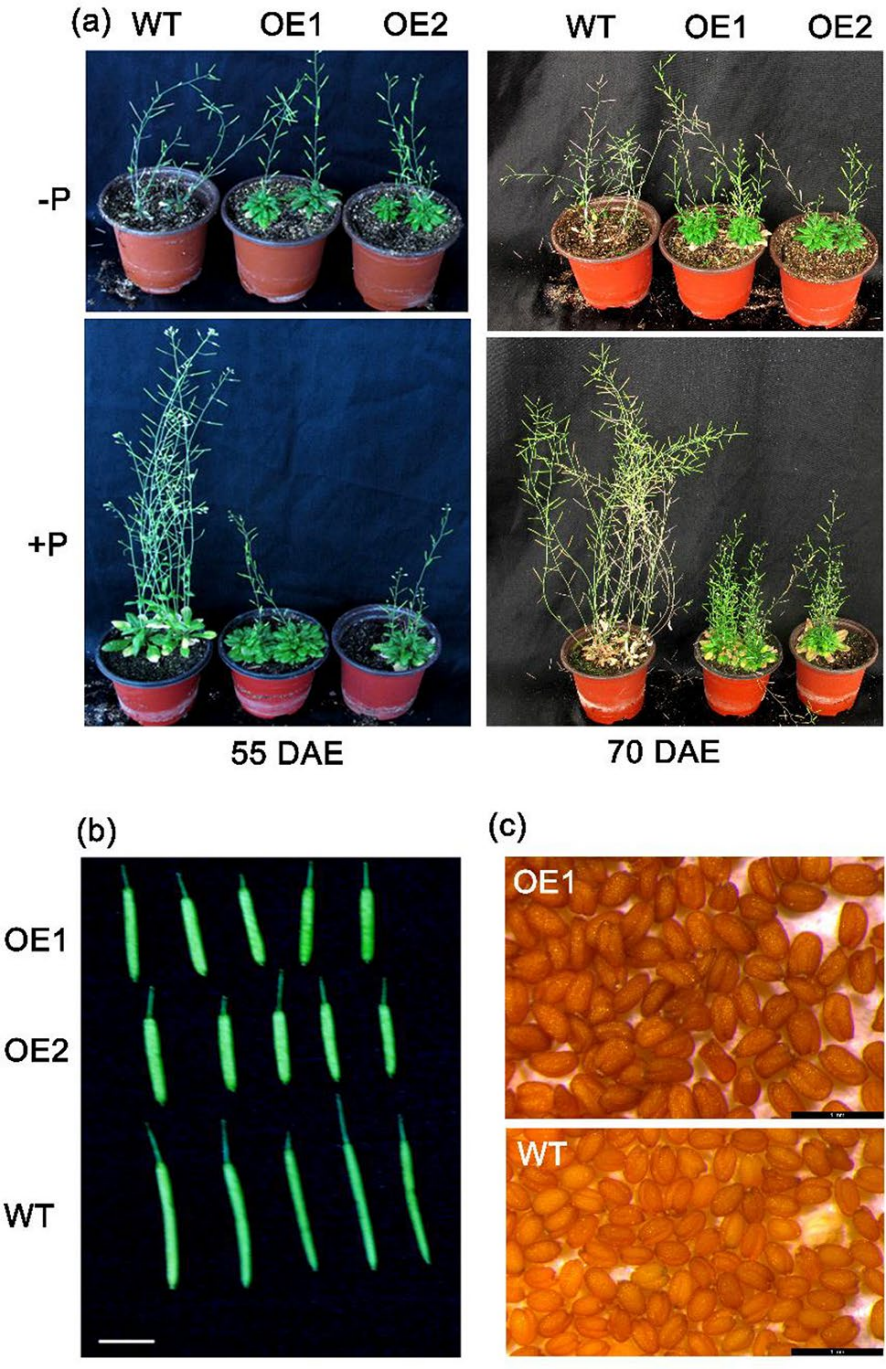
Phenotype of adult Arabidopsis plants grown under P-sufficient and P-deficient conditions. (**a**) The morphological phenotype of *GmmiR156b* overexpressing lines (OE1, OE2) and the wild type (WT) on 55 days and 70 days after emergence (DAE) under + P (P sufficient) and − P (P deficient conditions). (**b**) Siliques of WT and OE lines, bar = 1 cm. (**c**) Mature seeds of WT and OE lines, bar = 1 mm.

At the adult plant stage, the OE1 and OE2 lines still maintained a better tolerance to P deficiency than WT. Under − P condition, the WT plants grew poorly and the first senescent leaf appeared 55 days after emergence (DAE), whereas the OE1 and OE2 lines still grew vigorously and the leaves maintained green at least 70 DAE. Under normal conditions, the WT plants grew well until 70 DAE when all leaves became fully senescent, whereas the leaves of OE1 and OE2 were still green (Fig. 4a). Moreover, the total P content in roots of OE1 and OE2 was higher than that in WT under − P condition; however, there was no obvious difference under + P condition (Fig. 3d).

### Seed yield increased by *GmmiR156b* overexpression

**Figure 5:**
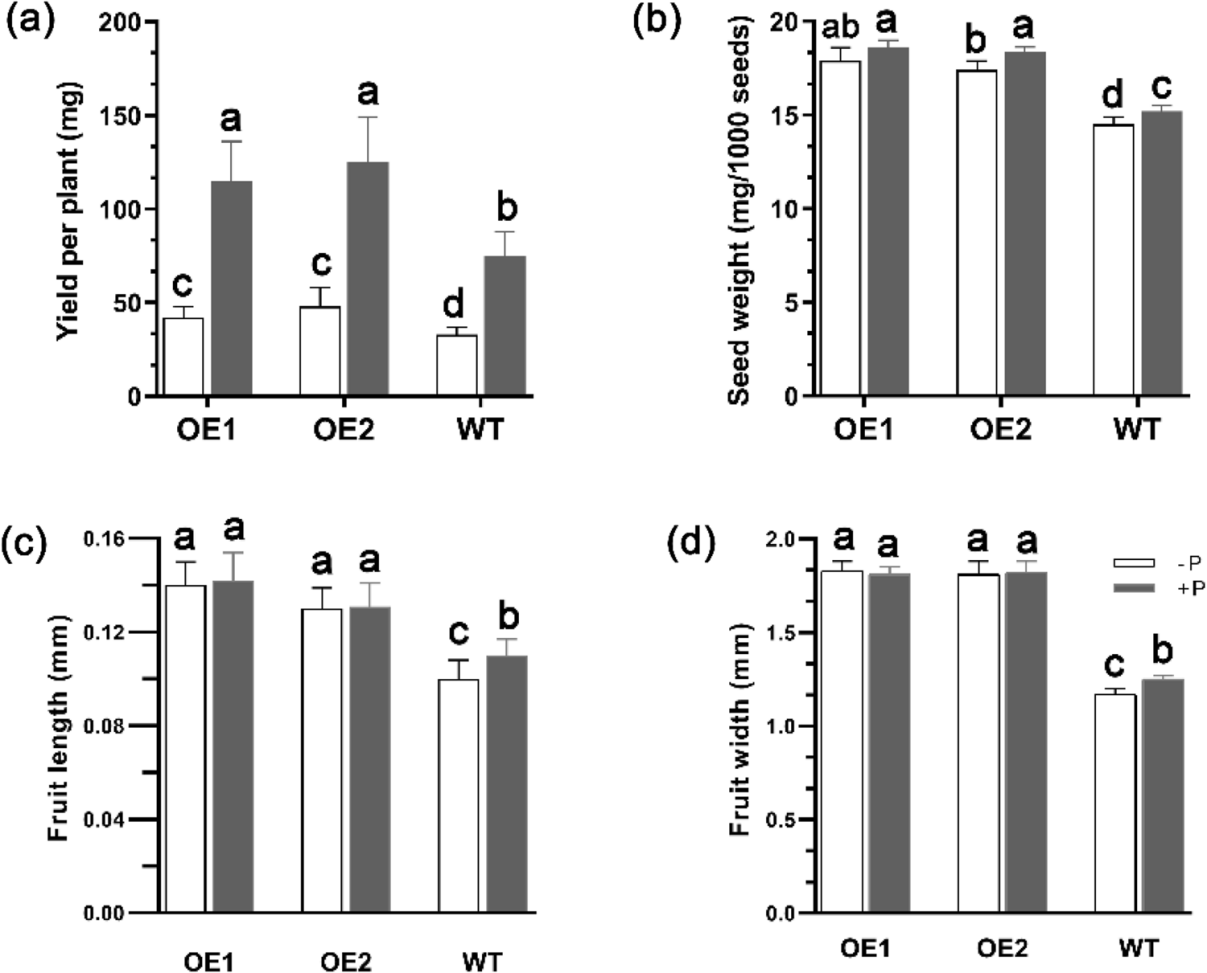
Yield traits in transgenic and wild-type Arabidopsis lines. (**a**) Seed yield of *GmmiR156b* overexpression (OE) and wild type (WT) plants under + P and − P conditions. (**b**) Seed weight of OE and WT plants under + P and − P conditions. (**c**) Fruit length of OE and WT plants under + P and − P conditions. (**d**) Fruit width of OE and WT plants under + P and − P conditions. Different letters above the bars indicate statistical significance (*P* < 0.05).

In normal growth conditions, the average number of rosette leaves in WT was 23.4 ± 3.5, which was much lower than the OE lines (44.4 ± 8.7) showing dwarfness; however, an opposite trend was found for the number of primary shoots (7.1 ± 1.3 versus 5.3 ± 1.2). Under − P condition, the WT plants were affected remarkedly but the OE lines were affected slightly (Fig. 4); the average seed yield per plant was 42–48 mg for the OE lines but only 33 mg for the WT (Fig. 5a), since the OE lines could grow for a much longer period (> 70 days) thus a better seed setting. It was noted that, although silique length was ~ 35% shorter in OE lines (Fig. 5c), the width of silique was much larger (Fig. 5d) and allowed for a bigger seed size (Figs. 4c and 5b).

### Expression of genes targeted by *GmmiR156b* in Arabidopsis

**Figure 6:**
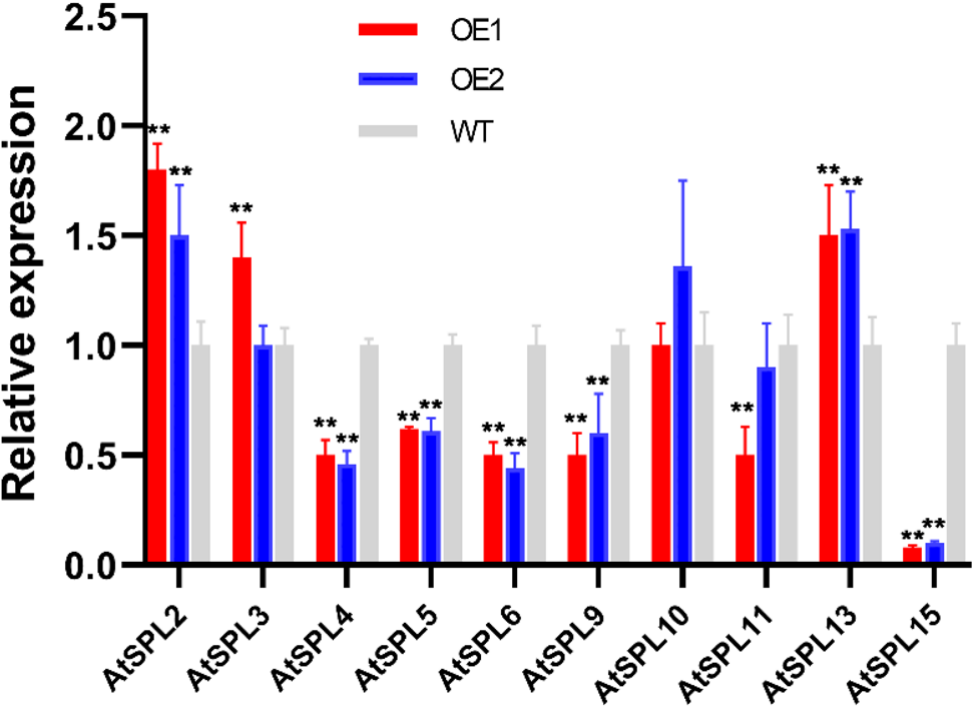
Transcriptional analysis of *AtSPLs* targeted by *GmmiR156b* in overexpression lines. Quantitative RT-PCR analysis was performed using RNA extracted from wild-type (WT) and overexpression lines (OE1 and OE2) for putative targets of *GmmiR156b* (i.e., *AtSPL2/3/4/5/6/9/10/11/13/15*). The relative expression level of *AtSPL*s in OE lines was normalized to that of the WT. *Tublin* was used as inner control for gene expression analysis. ** indicates a statistical difference between *AtSPLs* and *Tublin* at *P* < 0.01.

There are 10 members in the *SPL* gene family targeted by miR156 in Arabidopsis, seven of which can be grouped into three major clades based on their functions in the regulation of plant morphology and development, namely *AtSPL2/10/11*, *AtSPL9/15*, and *AtSPL6/13*^31^. To investigate which *SPL* genes (i.e., *AtSPL2/3/4*/*5/6/9/10/11/13/15*) are regulated by *GmmiR156b* in Arabidopsis transgenic lines, we detected the expression of the above ten *AtSPLs* in two *GmmiR156b* OE lines and WT plants by qRT-PCR. Six genes (*AtSPL4/5/6/9/11/15*) were down-regulated in at least one of the transgenic lines. Of these, the expression of *AtSPL15* was most affected and decreased by more than 90% (Fig. 6), suggesting that *AtSPL15* is the major target of *GmmiR156b*. In contrast, the expression of the other four *AtSPLs* (i.e., *AtSPL2/3/10/13*) in transgenic lines was equal to or higher than that in wild-type plants (Fig. 5), indicating that they are not the direct targets of *GmmiR156b*.

## Discussion

miR156 is a pivotal regulator for plant growth and development with pleiotropic effects. Several studies reported that miR156 is not only a key player in modulating a series of plant developmental processes such as branching, early flowering, fruit ripening, and cell proliferation but also in responses to environmental nutritional stresses by interaction with its targets, the *SPL* gene family^32–35^. Here we demonstrated for the first time, that overexpression of soybean homolog of miR156 in Arabidopsis could directly enhance the tolerance to P deficiency and seed yield under P deficit conditions, which is valuable for the development of efficient P-use soybean varieties in the future.

Plants usually cope with P deficiency by changing root architecture such as alteration of the root-to-shoot ratio, an increase of total root length, as well as lateral root lengths/numbers to improve the absorbance of P^33–35^. Lei et al.^19^ reported that miRNA156, together with its target SPL3, plays a pivotal role in responses to low P stress. They found that the transcript level of miR156 was significantly increased under low P condition but SPL3 was suppressed. Transgenic lines constantly expressing SPL3 grown under P-deficient conditions exhibited decreased rhizosphere acidification (thus closer to the optimal pH facilitating the release of insoluble P), which promoted P uptake. Moreover, a P transporter (i.e., Pht1;5) was found to be significantly activated via SPL3 and hence increased P uptake in the root^19^. In this study, however, we found that overexpression of *GmmiR156* didn’t alter the level of *AatSPL3* significantly, suggesting that *AatSPL3* is not the main target of *GmmiR156b* (Fig. 6). Instead, some other *SPL* genes (i.e., *AtSPL4/5/615*) were substantially suppressed, resulting in more efficient P uptake in the root, delayed flowering, and prolonged juvenal period. Therefore, we conclude that these four *SPL* genes (especially *AtSPL15*) are the main targets for *GmmiR156b*. In Arabidopsis, *SPL2/910/11/13/15* modulate a variety of biological processes in root and shoot development whereas *SPL3/4/5* predominately boosts floral induction and/or floral meristem identity^32^. *miR156* represses *SPL* gene expression by cleaving SPL transcripts and by promoting their translational repression^35^. So far, there are no reports demonstrating the importance of *SPL15* in P metabolite, and it is worth to further investigate the mechanism of the miR156-SPL15 module in the regulation of P uptake in soybean. Apart from miR156, many other miRNAs have been shown to take part in the P starvation response in soybean. For instance, Sha et al.^27^ identified 60 miRNAs in soybean via deep sequencing, which exhibited an altered gene expression pattern at least in roots or shoots under P stress conditions. Moreover, miRNA399-PHO2 modules are found to orchestrate the P homeostasis in Arabidopsis by mediating the loading of P in the xylem by the breakdown of PHO1^36,37^.

Rhizosphere acidification is frequently observed in response to low P stress, which expedites the discharge of P from soil and apoplasts^38^. *SPL3* overexpression plants displayed an increased level of P uptake because of decreased rhizosphere acidification^19^. In the present study, the adult plant of *GmmiR156* overexpression lines showed more tolerance to P starvation (Fig. 4). Higher P content was accumulated in shoots of transgenic lines which resulted in better growth of the transgenic lines than WT under P-deficient conditions. The senescence process of transgenic lines largely lagged behind the WT under both P deficient and P sufficient conditions (Fig. 4). One reason is likely that overexpression of *GmmiR156b* delayed the vegetative-to-reproductive phase transition^19^. Another explanation is that the continuous growing shoots, referred to as bushy architecture^39^, resulted in inconsistent maturity of the old and young shoots (Fig. 4).

We also observed that the primary root lengths of OE1 and OE2 seedlings are longer than that of WT, and P content is higher in shoots of OE1 and OE2 than in WT under low P stress conditions, suggesting that the overexpression of *GmmiR156b* might increase P uptake by promoting primary root growth of seedlings (Fig. 3). The result is similar to the report in maize that the length of 12-day-old seedlings was increased under low P conditions^40^. However, the ratio of root-to-shoot in OE1 and OE2 seedlings lines was decreased under both P deficient and P sufficient conditions. It might be attributed to the promoted shoot growth by overexpressing *GmmiR156*, which has been reported in torenia^39^, Arabidopsis^41^, tomato^42^, switchgrass^43^, and alfalfa^44^.

A group of genes control the two important transitions in shoot development and are coordinated by miR156 and its target *SPL* gene families^45^. Several studies reported that the pleiotropic effect of overexpressing miR156 includes the delay of juvenile-to-adult phase transition, more branches, smaller leaf size, and altered metabolism in plants^46–49^. Highly overexpressing of miR156b in transgenic poplar lines exhibited a globulous phenotype with many branches and leaves, markedly smaller leaf size, and the accumulation of reddish pigment^50^. Similar to the above reports, in the present study, overexpression of *GmmiR156b* showed increased seed yields and strong pleiotropic developmental phenotypes such as dwarfness, prolonged growth period, bushy shoot, and shorter silique length (Figs. 4 and 5). Our observation is also in agreement with the increase of soybean seed yield in *GmmiR156* overexpressed lines, by suppression of its target *GmSPL9*^51^. We also found that the length of silique was shorter in transgenic lines than in WT, which is reminiscent of the Arabidopsis *sk156* mutant in which expression of *miR156b* was enhanced^31^. Moreover, six *SPL* genes, namely *AtSPL4/5/6/11/15* were down-regulated in *GmmiR156b* overexpression lines (Fig. 6). Wei et al.^31^ reported that suppression of *SPL15* causes decreased silique size; *SPL4* and *SPL5* mainly control plant juvenile-to-adult transition; *SPL9* and *SPL15* regulate shoot maturation and leaf initiation, while *SPL11* modulate shoot maturation in the reproductive phase. Intriguingly, we found that *SPL15* was most suppressed in *GmmiR156b* OE lines, resulting in a longer growth period and delayed senescent progress than WT (Fig. 6). This information collectively indicates that the developmental morphology and increased P deficiency tolerance of *GmmiR156b* OE lines might be the consequence of the suppression of these target *SPL* genes.

In conclusion, here we showed that suppression of *SPL* genes, especially *AtSPL15* by *GmmiR156b* resulted in longer primary roots and higher P contents in the roots of seedlings. Overexpression of *GmmiR156b* also increased seed yields and exhibited strong pleiotropic developmental phenotypes under P-deficient conditions. These findings will deepen our understanding of the post-transcriptional regulation of gene expression under low P in higher plants and highlight the pivotal role of the miR156b-SPL15 module in responses to P starvation.

## Supplementary Information

Supplementary Table 1.

Supplementary Figure 1.

## Data Availability

The datasets used and/or analyzed during the current study are available from the corresponding author upon reasonable request. The DNA fragment containing soybean miR156b is deposited in NCBI under accession No. OP784762 (https://www.ncbi.nlm.nih.gov/).
