# Supplementary material for: Overexpression of soybean microRNA156b enhanced tolerance to phosphorus deficiency and seed yield in Arabidopsis
Source: Sci Rep. 2023 Jan 12;13:652. doi: 10.1038/s41598-023-27847-2 (PMC9837069; doi:10.1038/s41598-023-27847-2)
Supplement: Supplementary file 2 — Supplementary Figure 1. [file 41598_2023_27847_MOESM2_ESM.docx]

**Supplementary Figure 1**

**Verification of transgenic events and quantification of *GmmiR156b* expression in Arabidopsis seedlings**. (a) PCR confirmation of positive transgenic Arabidopsis plants carrying *GmmiR156b* by gene-specific primers. (b) RT-PCR analysis of *pri-GmmiR156b* in the leaf of the wild type (WT) and eight independent transgenic lines. Lanes 1-8, transgenic lines; lane 9, the WT. M, DNA marker DL2000.

**1 2 3 4 5 6 7 8 M**

1. **miR156B**


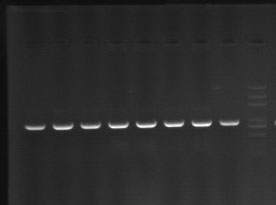


**-201 bp**

**Tublin**

**1 2 3 4 5 6 7 8 9 M**

**Pri-miR156b**

**1 2 3 4 5 6 7 8 9 M**


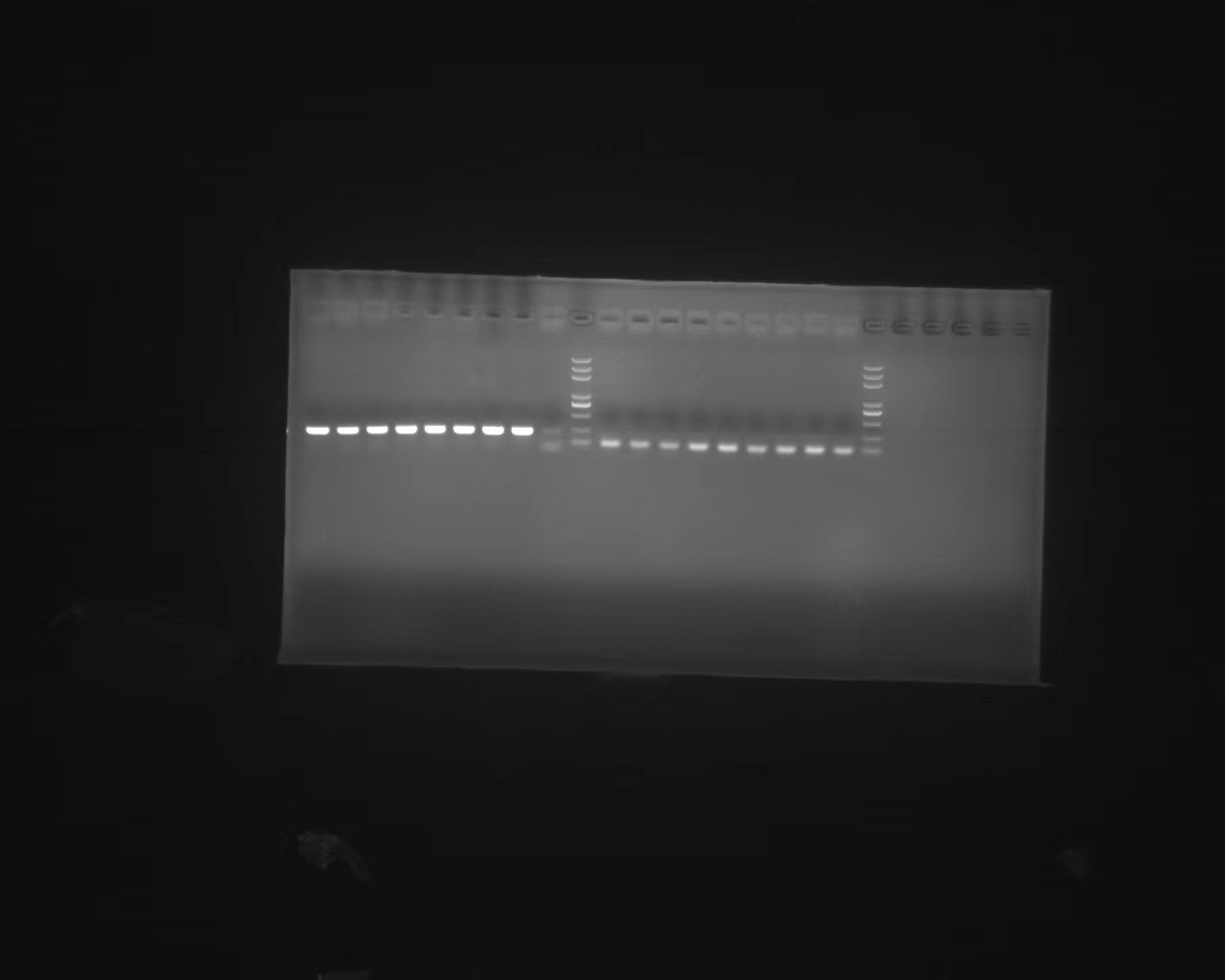


**-201 bp**
